# An automated or semi-automated identification system using venation pattern to delimit Indian leaf drugs: A proposal

**DOI:** 10.4103/0974-8490.75461

**Published:** 2010

**Authors:** A.B.D. Selvam

**Affiliations:** *Pharmacognosy Section, Botanical Survey of India, Howrah - 711 103, India*

**Keywords:** Automated, botanist, identification, Indian leaf drugs, pharmacognosist, pharmacognosy, semi-automated, venation pattern

## Abstract

About 7,200 medicinal plants are known to occur in India, of which, the leaves of a few hundred plants have medicinal properties. Identification of leaf drugs using venation is considered as one of the most reliable and convenient methods. Leaf identification by mechanical means may often lead to wrong identification. Due to the growing volume of illegal trade/malpractice in the crude drug industry on the one hand and lack of sufficient experts on the other, a much faster, convenient and reliable method is mandatory for the identification of Indian leaf drugs. Therefore, a new automated or semi-automated identification system based on venation pattern is inevitable for the present day condition to identify and authenticate the leaves of Indian medicinal plants.

## INTRODUCTION

Nature is an everlasting mystery to mankind. Man has been taking all possible efforts to understand nature and unravel its facts ever since human civilization. During the last few centuries, scientists across the globe have mastered the art of understanding the mysteries and utilizing the same for the betterment of humanity.

Altogether about 4,10,800 plant species are known to exist presently in the world. Many plant species are yet to be discovered by the botanists. India has been recognized as one of the 17 mega diverse countries and one of the mega gene centers of the world. In India, there are ca. 46,340 species of plants and many more are yet to be identified and described,[1] of which about 7,200 plant species are reported to have medicinal properties,[2] out of which, the leaves of a few hundred plants have therapeutic properties.

Each and every plant species has got its own individuality and uniqueness within it which is revealed through various characters embedded in it. The leaves of flowering plants show highly diverse and elaborate venation patterns and are considered as one of the constant diagnostic characters in the identification of plant species. In addition to venation, the other taxonomic characters of a leaf such as size, shape, margin, base, apex, stomata and palisade ratio are also taken into due consideration to identify the plant species.

Venation is the pattern of veins in the blade of a leaf. The veins consist of vascular tissues which are vital for the transport of food and water, which also give mechanical support to the leaves. The leaf has a midrib (midvein), which further branches into a number of lateral veins and marginal veins. The venation is more visible in the lower surface (adaxial) of the leaves than the upper surface (abaxial) of leaves.

The venation pattern has been broadly divided into two types, viz. parallel venation in the case of leaves of monocotyledons and reticulate venation (netted venation) in the leaves of dicotyledons. The venation patterns are being determined in terms of veinlets (vein-islets) and veinlet termination numbers located per square millimeter of the leaf surface midway between the midrib and the margin. A vein termination is the ultimate free termination of veinlet. It has been shown that the number of veinlets/vein-islets per unit area of leaf surface is constant for any given species of plant and can be used as a character for the identification of species.[3]

Identification of plants has been based largely on features of the reproductive parts such as flowers and fruits. In the absence of such parts, the drug plants, particularly the leaf drugs of dicotyledons are being identified by taxonomists, anatomists and pharmacognosists using venation pattern and other leaf features.

## EXPLICATORY EXAMPLES

The leaf architecture working group at the Smithsonian Institution has prepared a manual, which provides unambiguous and standard botanical terms for describing leaf form and venation with illustration, particularly of dicotyledonous plants. Further, this manual provides a template and set of instructions and descriptive information that can be entered into a standardized database of fossil and existing leaves.[4]

One of the modern methods for obtaining the venation patterns of the leaves is the preparation of X-ray images/photographs of fresh and dry leaves. Based on this technique, the venation details of about 1056 Australian tropical rainforest tree species have been prepared using low-voltage X-ray photographs, which allow visual confirmation of identifications made with the computer key.[5]

The Indian leaf drugs have already been studied pharmacognostically.[6] A field key using vegetative characters, mainly exudation, armature and leaf characters such as arrangement, size, shape, and venation pattern has been prepared to identify the trees and lianas of the evergreen forests of the Western Ghats of India.[7] Tree ID has been designed to identify the trees of Kerala by selecting a few easily observable/recognizable key characters such as bole, leaf form, leaf margin, exudation, nerves etc.[8]

Identification of leaf drugs using venation is still considered as one of the most reliable and convenient means for distinguishing them from allied species or superficially resembling plant species. Leaf identification by visual and manual/mechanical means may often lead to wrong identification. Due to the growing volume of illegal trade and malpractice in the crude drug industry on the one hand and lack of sufficient experts on the other, a much faster, convenient and reliable method is mandatory for the identification of Indian leaf drugs.

Nowadays, fingerprints of men are very much used as an identification tool using automated system. In the same way, veins of drug plants can be used as a scientific tool to identify/authenticate them in their crude form. Following the above-mentioned protocols, a new automated or semi-automated identification system using venation pattern, named Venation Image Database System (VIDS) may be planned/designed for Indian medicinal plants (drug plants) in general and Indian leaf drugs in particular. In this system, in addition to the venation pattern, other taxonomic characters of a leaf such as shape, size, margin, base, apex and venation can also be taken into due consideration to identify/authenticate the Indian leaf drugs. The data of leaf architecture can be prepared from X-ray photographs of fresh and dry leaves or by means of digitization. Identification by automated or semi-automated means would be much faster and accurate than by mechanical means.

## CONCLUSION

The living creatures resemble one another in many features. One such peculiar resembling character is, lines on the palms of human hands and veins on the leaves of plants. A theme phrase weaved by the author in this context has been depicted hereunder.

“As a palmist could read the lines on the palms of hands to predicts one’s life

So as a botanist could read the veins on the leaves of plants to identify the plants”

In the light of the above mentioned factors, it is suggested that the competent and concerned authorities should come forward to constitute a committee comprising experts (taxonomists, anatomists, pharmacognosists, computer software professionals etc.) to design and develop a new automated or semi-automated identification system based on leaf features, especially venation pattern, to identify and authenticate Indian medicinal plants in general and leaves of medicinal plants in particular.
